# Supplementary material for: Functional Characteristics of Diverse PAX6 Mutations Associated with Isolated Foveal Hypoplasia
Source: Genes (Basel). 2023 Jul 21;14(7):1483. doi: 10.3390/genes14071483 (PMC10379490; doi:10.3390/genes14071483)
Supplement: Supplementary file 1 [file genes-14-01483-s001.zip › genes-2494641-supplementary.pdf]

Fig. S1

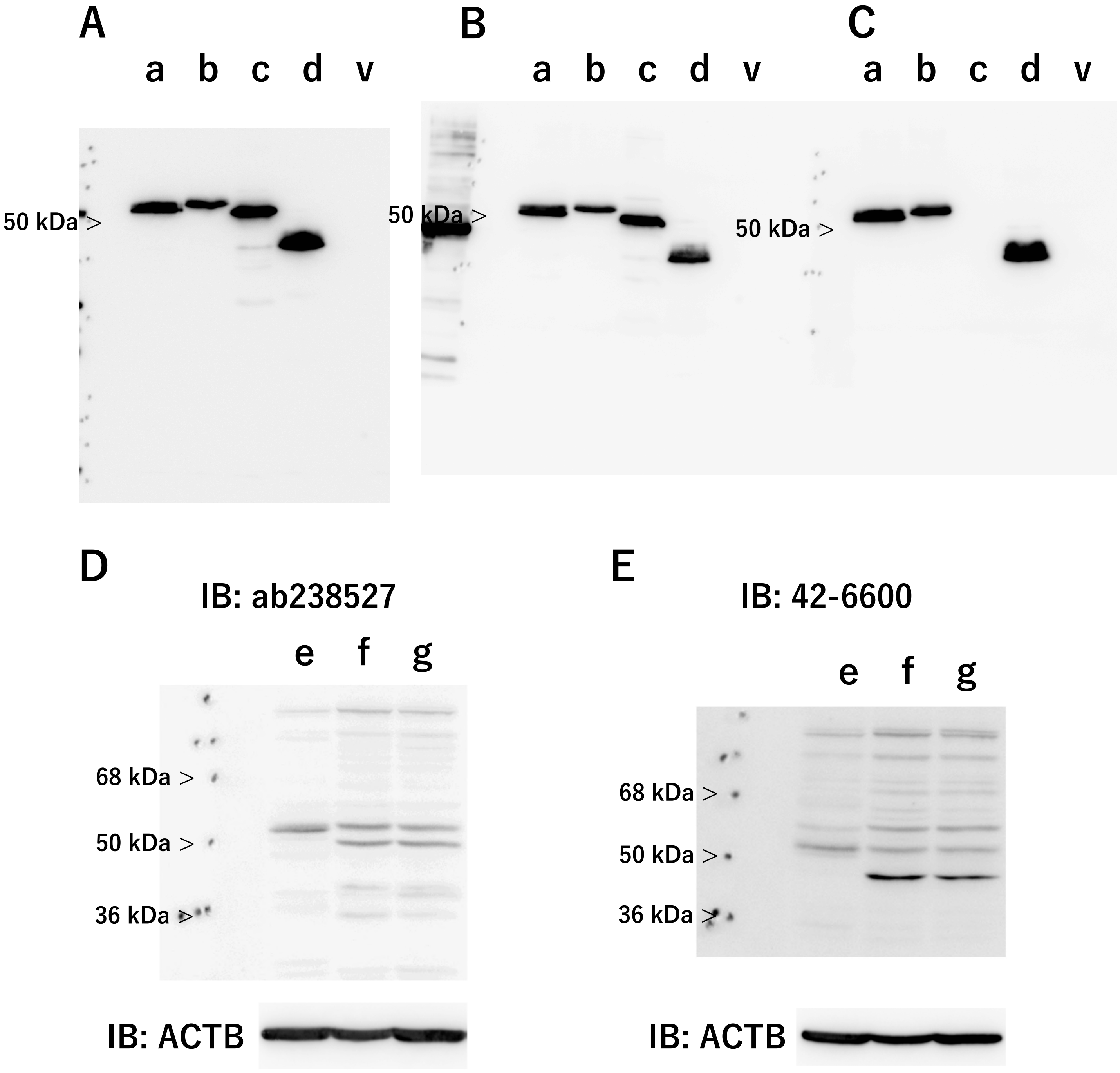
A
a b c d v
50 kDa >
B
a b c d v
50 kDa >
C
a b c d v
50 kDa >
D
IB: ab238527
e f g
68 kDa >
50 kDa >
36 kDa >
IB: ACTB
E
IB: 42-6600
e f g
68 kDa >
50 kDa >
36 kDa >
IB: ACTB

F

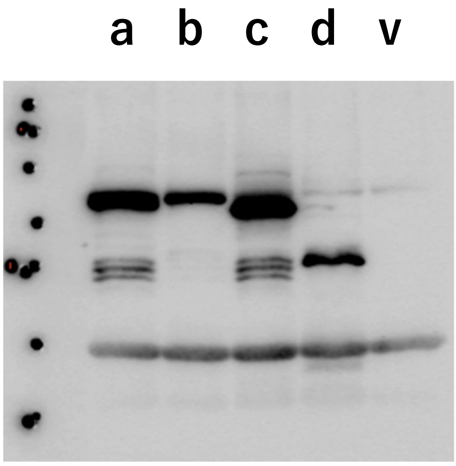
a b c d v

G

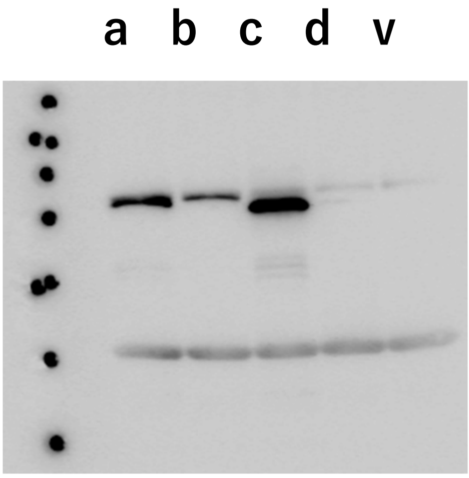
a b c d v

H

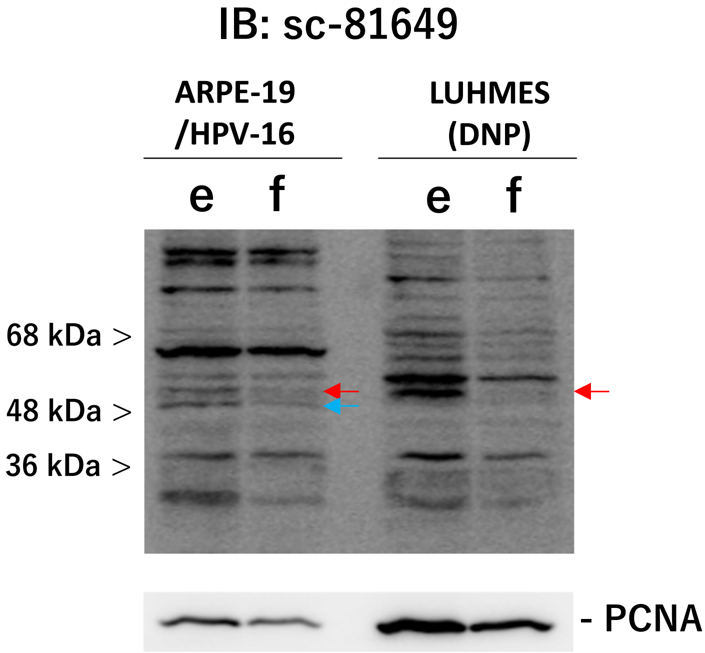
IB: sc-81649
ARPE-19
/HPV-16
LUHMES
(DNP)
e f e f
68 kDa >
48 kDa >
36 kDa >
- PCNA

I

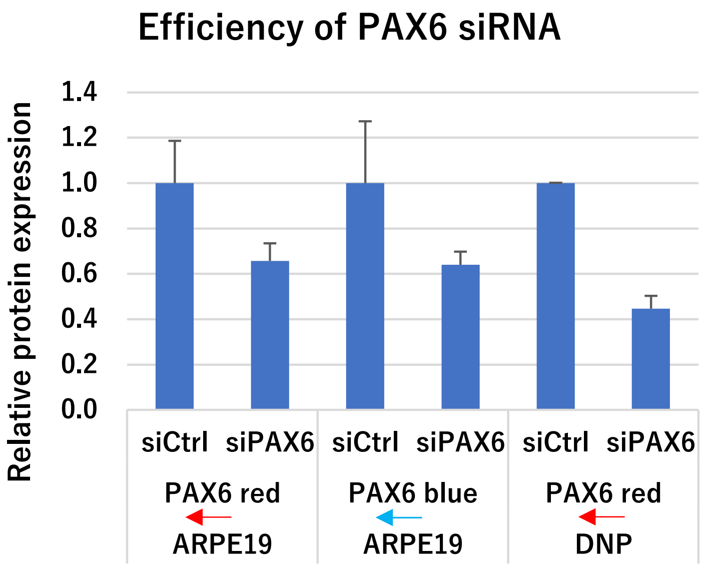
Efficiency of PAX6 siRNA
Relative protein expression
1.4
1.2
1.0
0.8
0.6
0.4
0.2
0.0
siCtrl siPAX6 siCtrl siPAX6 siCtrl siPAX6
PAX6 red
ARPE19
PAX6 blue
ARPE19
PAX6 red
DNP

Fig. S1 Evaluation of sensitivity and specificity of commercially available antibodies.

Method: Flag-PAX6 isoforms (isoform a; NM_000280, isoform b/5a; NM_001604, isoform c; NM_001310159, isoform d; NM_001310161) and vector alone (v) were transiently expressed in PC3 cells and immunoprecipitated with an anti-Flag antibody. The transferred membranes were treated with anti-Flag antibody (Sigma; M2-HRP) (A), anti-PAX6 antibody (Abcam; ab238527) and anti-rabbit IgG antibody conjugated HRP (B), anti-PAX6 antibody (Invitrogen; 42-6600) and anti-rabbit IgG antibody conjugated HRP (C). (D)(E) Whole cell lysate (50 µg) of ARPE19/HPV16 (e), LUHMES (f) and PC3 (g) cells were subjected with SDS-PAGE. Each membrane was blotted with anti-PAX6 antibodies (ab238527 and 42-6600) and anti-rabbit IgG antibody conjugated HRP. These transfer membranes were also blotted with anti-ACTB antibody conjugated HRP (Sigma; A3854) for internal control. (F) Similar to (A), Flag-PAX6 isoforms a, b/5a, c, d and vector alone (v) were transiently expressed in PC3 cells and immunoprecipitated with an anti-Flag antibody. The transferred membrane was treated with anti-Flag antibody (Sigma; M2-HRP), and also treated with anti-PAX6 antibody (Santa Cruz; sc-81649) and anti-mouse IgG antibody conjugated HRP (G). (H) ARPE19/HPV16 and LUHMES cells were transfected with siRNA for control (e) and PAX6 (f; Silencer® Select s529239, Thermo Fisher Scientific Inc.). After 48 hours, cells were collected and separated into nucleoplasm and cytoplasm. Each nuclear protein (50 µg) was subjected with SDS-PAGE and blotted with anti-PAX6 antibody (Santa Cruz; sc-81649) and anti-mouse IgG antibody conjugated HRP. This transfer membrane was also blotted with anti-PCNA antibody (Santa Cruz; sc-56) and anti-mouse IgG antibody conjugated HRP for internal control. (I) In (H), the decreased signal around the predicted molecular weight of PAX6(5a) in (f) compared to (e) was quantified (n=2). siCtrl and siPAX6 indicate siRNA for control and that for PAX6, respectively.

Result: Three anti-PAX6 antibodies, ab238527, 42-6600 and sc-81649, detected the Flag-PAX6 isoforms b/5a. However, no signal of PAX6 (5a) was observed for antibodies (ab238527 and 42-6600) in ARPE19/HPV16 and LUHMES cells compared to PC3 cells (D and E). On the other hand, using the anti-PAX6 antibody (sc-81649), several signals whose expression was reduced by siPAX6 treatment were observed, suggesting that PAX6(5a) may be among these signals (H and I). However, the PAX6(5a) signal with anti-PAX6 antibody (sc-81649) has low specificity, so it was judged difficult to identify it.
